# Spatial, temporal, and demographic nonstationary dynamics of COVID-19 exposure among older adults in the U.S.

**DOI:** 10.1371/journal.pone.0307303

**Published:** 2024-08-22

**Authors:** Qian Huang

**Affiliations:** Center for Rural Health Research, College of Public Health, East Tennessee State University, Johnson City, Tennessee, United States of America; Villanova University, UNITED STATES OF AMERICA

## Abstract

This study examines demographic disparities in COVID-19 exposures across older adults age 60–79 and older adults age 80 and over, and explores the factors driving these dynamics in the United States (U.S.) from January 2020 to July 2022. Spatial clusters were identified, and 14 main health determinants were synthesized from 62 pre-existing county-level variables. The study also assessed the correlation between these health determinants and COVID-19 incidence rates for both age groups during the pandemic years. Further examination of incidence rates in relation to health determinants was carried out through statistical and spatial regression models. Results show that individuals aged 80 and over had much higher hospitalization rates, death rates, and case-fatality rates in 2020–2022. Spatial results indicate that the geographical cluster of high incidence rates for both groups shifted from the Midwest at the beginning of the pandemic to the Southwest in 2022. The study revealed marked spatial, temporal, and demographic nonstationary dynamics in COVID-19 exposures, indicating that the health effects of contextual factors vary across age groups. COVID-19 incidence rates in older adults were strongly influenced by race, healthcare access, social capital, environment, household composition, and mobility. Future public health policies and mitigations should further their efforts by considering temporal and demographic nonstationarity as well as local conditions.

## 1. Introduction

Although absolute COVID-19 risks are still uncertain, studies identify a demographic risk gradient [1, 2]. Older adult patients have been recognized as one of the most vulnerable groups since the beginning of the pandemic [3]. Incidence studies in various locations and settings found a higher proportion of older adults within the overall patient population [1]. In particular, these individuals, when residing in long-term care, accounted for 30–70% of COVID-19 mortalities during the initial surge of the disease in high-income countries [4].

Current evidence suggests that social, behavioral, environmental, healthcare access, political contexts, and geography are related to the risk of COVID-19 infections and its severity, and that these impacts vary across age groups [4–6]. Previous research investigating the linkage between health determinants and incidence rates typically centered on one specific set of determinants. For instance, the interplay between COVID-19 outcomes and contextual factors like public health policies and nursing home settings has been studied in detail [7–9]. In a parallel vein, quantifiable metrics such as race and ethnicity, poverty, marital status, and education have been studied to highlight the social determinants of health and their associated vulnerabilities [10, 11]. However, the difference in the variables’ effects on health outcomes within different age groups, especially among older adults, has received relatively little attention.

Using a large dataset of over 13 million older adult COVID-19 patients in the U.S. between January 2020 to July 2022, this study explored differences in COVID-19 exposures across two older adult groups–YE, age 60–79, and SR, age 80 and over–and the spatial variability in the driving factors producing such outcomes. The driving factors include community-level political, social, behavioral, environmental, and healthcare access contexts in the U.S.

## 2. Materials and methods

### 2.1 Community-based COVID-19 spatial disparity conceptual model

**Fig 1:**
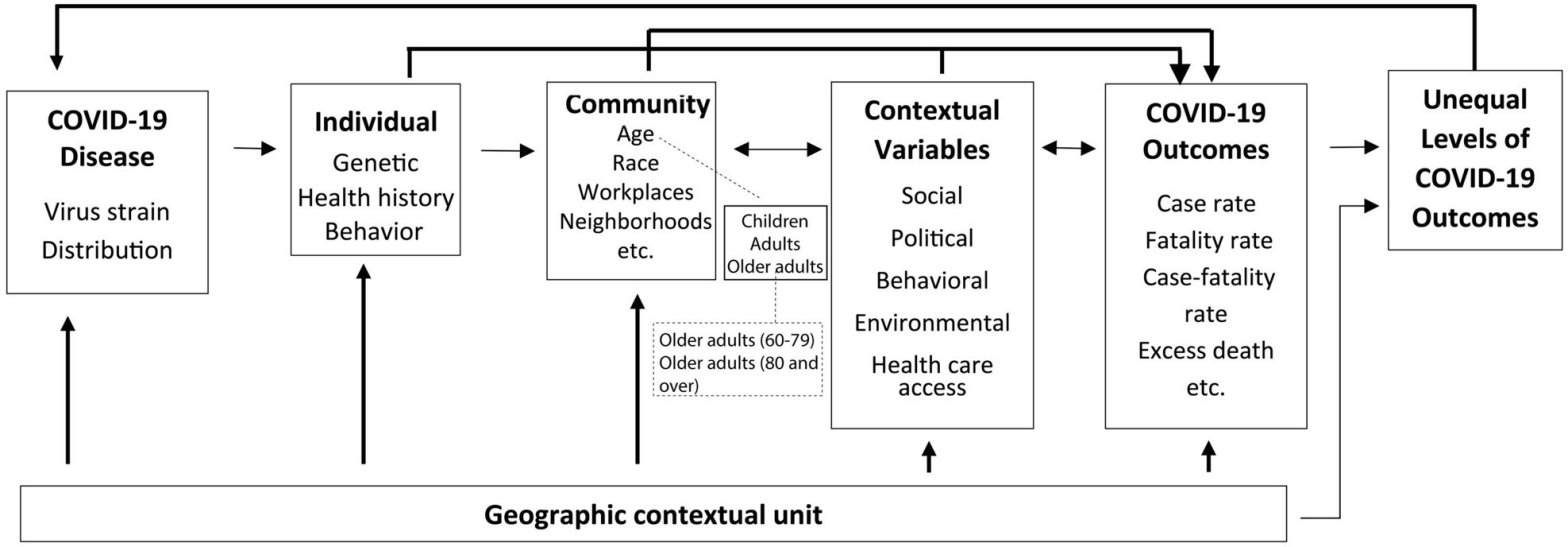
Community-based COVID-19 spatial disparity model (Adapted from Huang [15]).

While comprehensive models have been constructed to identify risk factors contributing to disease burden disparities [12–14], they do not holistically address the intricate spatial and demographic dimensions that shape the disparities observed in COVID-19 outcomes. These dimensions span political, social, behavioral, environmental, and healthcare access factors. This study expands the conceptual framework developed by Huang [15], in which genetic factors, health history, and behaviors work in tandem with broader contextual factors to affect individual health outcomes. Fig 1 extends the community-based COVID-19 spatial disparity model by categorizing the older adult group into subgroups: older adults age 60–79 and older adults age 80 and over. Community classification by age group directly impacts COVID-19 outcomes. Simultaneously, contextual variables intertwine with individual-level and age-specific community-level factors, modifying social, political, behavioral, environmental, and healthcare access landscapes, thereby influencing the distribution of COVID-19 outcomes. The outcomes themselves can also reciprocally affect these variables—for instance, amplifying the adoption of telehealth services or mobile clinics in response to the disease. Central to this dynamic is the geographic contextual unit, which essentially frames the observed disparities in COVID-19 outcomes. These disparities can, in turn, evolve when faced with emergent factors, such as the introduction of new viral strains.

### 2.2 COVID-19 surveillance data

The COVID-19 case surveillance data used for this research was requested from the Centers for Disease Control and Prevention (CDC) on August 1st, 2022. This dataset encompasses patient-specific records of COVID-19 cases relayed to the CDC. It details patients’ demographics, county of residence, potential exposure history, severity and outcomes of the disease, as well as pre-existing medical conditions for the period from January 2020 to July 2022 [16]. Data reporting is voluntary and varies by state, so the true number of cases may be underestimated. The outcomes, including hospitalization and death, may be incomplete because they might not yet have been known at the time of reporting or a patient’s condition might have changed after case data submission without being updated [16]. The patients are categorized into YE group (age 60–79) and SR group (age 80 and over).

### 2.3 Disparity assessment

The study aggregated the total number of newly infected incidences in a given county over the previous 7 days (including the given day) starting from January 1st, 2020, and calculated the daily average during this time period, and then converted this number to the average daily fraction of the population in each county that was infected during this week by dividing it by the county population to calculate the 7-day moving average. Subsequently, the 7-day moving average of COVID-19 daily new incidences, deaths, and hospitalizations per 100,000 population from January 2020 to July 2022 (930 days) for two age groups were calculated and compared.

To discern the COVID-19 outcome differences across age groups over the years, a one-way ANOVA was applied to compare 6 groups: YE in 2020 (YE20, n = 366 days), 2021 (YE21, n = 365 days), 2022 (YE22, n = 199 days), and SR in 2020 (SR20, n = 366 days), 2021 (SR21, n = 365 days), and 2022 (SR22, n = 199 days).

For spatial disparities, the COVID-19 incidence rates (incidences per 100,000 population) were assessed. Incidence rates of the six groups (YE20, YE21, YE22, SR20, SR21, and SR22) were visualized using ArcGIS Pro 3.0.3. Moran’s I analyses, both Global and Anselin Local, were conducted to identify spatial clusters of counties with high or low values and spatial outliers.

### 2.4 Determinants of health

The distribution and severity of COVID-19 exposure were mainly, but not exclusively, associated with contextual variables such as political, social, behavioral, and environmental factors, as well as access to healthcare [17, 18]. To examine the impacts of health determinants on COVID-19 exposure risks, the study included 62 indicators that characterized the broader dimensions of policies and political affiliation, socioeconomic, behavioral, perceptual, and comorbidity, environment, and healthcare provider and access in all 3,142 U.S. counties after testing for multicollinearity. The variable name, description, data source, time frame, and rationale for inclusion are reported in S1 Table. The data and method are elucidated in further detail below.

#### 2.4.1 Policies and political affiliation

Policy responses such as limiting travel, mask mandates, and physical distancing effectively controlled the spread of the virus [19–21]. Trust in government and government corruption had significant correlations, positive and negative respectively, with lower infection rates [22]. Research showed that the pandemic killed more Republican voters due to their opposition to mask mandates and vaccinations [23]. To evaluate the effectiveness of COVID-19 policy interventions, this study utilized data from four distinct sources, encompassing stay-at-home orders, emergency declarations, mask mandates, and closures of daycares and businesses from early 2020 to July 2022 (S1 Table). The study adopted a binary coding approach to represent the enactment (coded as 1) or non-enactment (coded as 0) of these mitigation measures, both at the county and state levels. In cases where a particular preventive measure was mandated statewide, each county within that state was assigned a 1; a 0 was assigned if the county deviated from the state mandate. Cumulative scores, reflecting the totality of county and state interventions, ranged from 0 to 4, and when combined for all years under consideration, this range extended from 0 to 8. The percentage of votes for the Democratic Party, from the MIT 2020 Election Data and Science, was used to capture political affiliation.

#### 2.4.2 Socioeconomic

Killerby et al. [24] suggested that socioeconomic factors, including employment as essential workers, economic instability, and higher numbers in households, contribute to a higher risk of COVID-19 infection and hospitalization. Low social status is perceived as a factor in not following the social distancing order and mask mandates [25]. The socioeconomic data incorporated 18 variables to capture the pre-existing county socioeconomic landscapes. The variables from American Community Survey 5-Year Data (2016–2020) covered poverty rates, median household incomes, percentages of renters, marital status, gender distribution, racial demographics, English speaking skills, proportions of female-led households, households with children, educational achievements, population densities, and households lacking vehicular access. To complement this data set, unemployment rates, income disparity indices, rates of population growth, health insurance coverage, the prevalence of healthcare-associated professions, and the proportion of residents with disabilities were also integrated (S1 Table).

#### 2.4.3 Behavioral, perceptual, and comorbidity

Behavior, perception, and comorbidities significantly influence the risk and severity of COVID-19 infections. Patients with comorbidities, including obesity, cardiovascular diseases, hypertension, diabetes, malignancy, renal diseases, and HIV, are at greater risk of severe illness and hospitalization from COVID-19 [26]. The HIV-positive population was particularly vulnerable during the pandemic, facing challenges in receiving treatment and monitoring [27]. Religious practices that require congregating can heighten the risk of COVID-19 infections [28], while increased full vaccination rates were moderately and negatively correlated with case and fatality rates [29]. Behavioral, perceptual, and comorbidity data incorporated sixteen variables. COVID-19 vaccination rates, showcasing cumulative percentages of fully vaccinated individuals (primary series) from 2020–2022, were sourced from the CDC as of August 2022. Comorbidity indicators, including conditions like hypertension, cardiovascular diseases, and diabetes, were derived from the CDC PLACES and BRFSS County Health Rankings. Religious affiliations came from the US Religious Census, while data on alcohol consumption, physical inactivity, and social associations were obtained from the BRFSS County Health Rankings.

#### 2.4.4 Environment

The COVID-19 pandemic underscored the importance of green space, living environment, safety, and air quality in bolstering physical and mental well-being. Green spaces, like parks, have become one of the only sources of resilience amidst the COVID-19 pandemic because of their positive effects on physical, psychological, and spiritual wellness [30]. Long-term exposure to PM_2.5_ and toxic substances can increase susceptibility to COVID-19 [31, 32]. Additionally, Stafoggia et al. [33] reported that increases in PM_2.5_, PM_10_, and NO_2_ levels were associated with higher COVID-19 case-fatality rates, particularly among older adults. These associations were more pronounced during the periods of February–June 2020 and December 2020–June 2021 [33]. Thus data included thirteen variables, covering access to parks, food environment, school, the amount of emissions from Toxics Release Inventory (TRIs), Ozone Days, rural population, as well as workplace and grocery mobility. Details of these data sources can be found in the S1 Table.

#### 2.4.5 Healthcare provider and access

Barriers to timely healthcare access can exacerbate COVID-19 hospitalization and fatality rates, particularly among minority ethnic groups [34, 35]. Mobile clinics provide a flexible solution in areas with inadequate healthcare infrastructure, effectively bridging gaps in the healthcare safety net for displaced and isolated individuals [36]. Healthcare provider and access data were sourced from the Health Resources & Services Administration (HRSA), HIFLD, Mobile Health Map, and FEMA. The data included the standardized numbers of healthcare providers (such as doctors, internal MDs, and primary care providers), facilities (including hospitals, pharmacies, nursing homes, emergency departments, hospital beds, and mental health centers), services (like mobile vans and telehealth) and federal support.

#### 2.4.6 Principal component analysis (PCA)

Missing values at the county level was replaced by the state average or national average if the state value was missing. This process was performed in Python 3.8.8. Then this study employed PCA to simplify the dataset’s dimensionality, utilizing a varimax rotation with 100 iterations and the Kaiser Criterion with 100 iterations (eigenvalues over 1) to select components. This reduces the likelihood of variables heavily loading onto multiple factors. This method identified variables that are closely aligned and transformed the original variables into multi-dimensional health components. PCA allows for a robust and consistent set of variables that can be tracked over time to evaluate any changes. This technique also facilitates replication of the analyses across various spatial scales, enhancing the efficiency of data compilation [37]. IBM SPSS Statistics 28 was used for this computation.

### 2.5 Statistical analysis and models

The relationships between COVID-19 incidence rates and determinants of health components were tested using Spearman’s rho correlation coefficients for both YE and SR groups during the pandemic. To further estimate the incidence rates with the components, an ordinary least squares (OLS) model was initially conducted to provide baseline regression models between incidence rates and determinants of health components used as correlates. To assess the necessity of integrating a spatial component into the regression model, the residuals from this model underwent a spatial dependence analysis using the global Moran’s I statistics, following the decision-making framework proposed by Luc Anselin [38]. Subsequently, Lagrange multiplier tests determined whether the spatial lag or error model was the optimal spatial model to adopt [38]. Moreover, the residuals, Akaike information criterion (AIC), Wald Statistics, log-likelihood, and R^2^ were used to assess the quality of the final model. The procedure was repeated for YE and SR separately for all pandemic years. The analyses were conducted with IBM SPSS Statistics 28 and GeoDa 1.20.0.20.

## 3. Results

### 3.1 Temporal dynamics of COVID-19 exposure disparities in the older adult groups

As of July 18, 2022, there were 82,422,585 COVID-19 cases reported in the CDC surveillance system, and 14,164,555 cases were of individuals age 60 and over. After excluding the records that were in the U.S. territory or with missing time/location (2.21% of 14,164,555 cases), 13,850,874 older adult patients (age 60+) were included in this study with age, infected time, and residence information distributed throughout all 50 U.S. states. Of the total, 11,395,362 (82.3%) patients were in the YE (60–79) group, and 2,455,512 (17.7%) patients were in the SR (80 and over) group.

**Fig 2:**
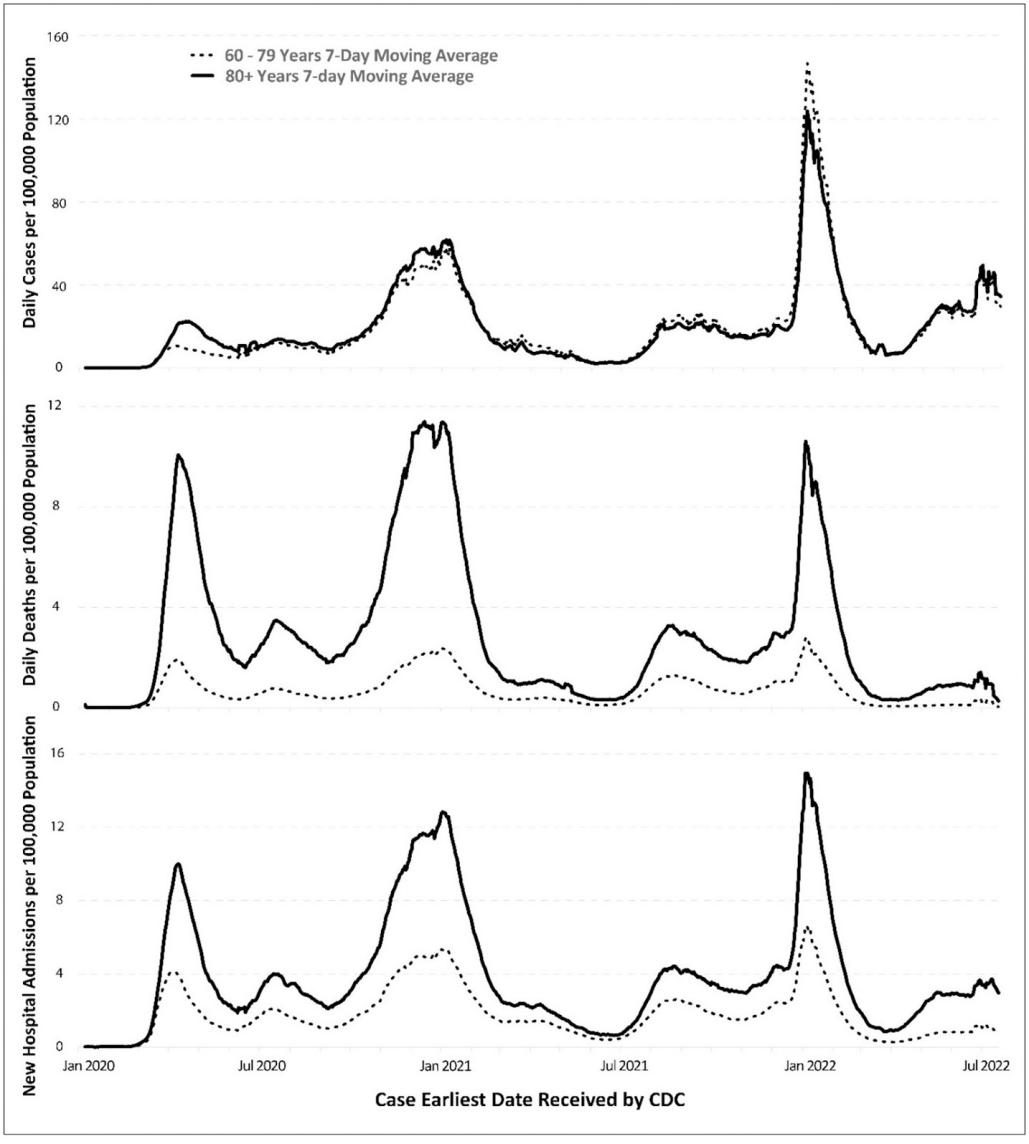
Daily COVID-19 cases (top), deaths (middle), and hospital admissions (bottom) per 100, 000 population in older adult groups in the U.S. from Jan 2020—Jul 2022 (7-day moving average).

The first wave of new cases, hospitalizations, and deaths occurred in the spring of 2020 (Fig 2). The YE group had somewhat similar incidence rates as the SR group, but the former had much lower death rates and hospitalization rates. The second COVID-19 wave appeared between December 2020 and February 2021, when the initial virus mutated with the new Alpha variant and spread. In January 2022, the incidence rates rapidly increased in the younger group and were higher than in the older group. This was the third wave of the pandemic when the Omicron variant replaced the Delta variant and became the variant of concern in the U.S. Nevertheless, the daily death rates and hospital admissions of SR were still much higher than those of YE.

Specifically, the incidence rate of COVID-19 in SR was 1.24 times higher than in YE in 2020 (Table 1). However, the incidence rates in YE increased in 2021 and 2022 and were slightly higher than those in SR. Nonetheless, SR had much higher hospitalization rates and death rates during the study period, with hospitalization rates being twice as high and death rates being 3–5 times higher than YE. In addition, the case-fatality rates were much higher for the older group.

**Table 1:** Descriptive results of COVID-19 outcomes of YE (60–79) and SR (80 and over) between January 2020 to July 2022.

Age Groups	Counties	Incidence	Incidence rates	Hospitalized*	Hospitalization rates	Death *	Death rates	Case fatality rate (%)
**2020**								
**YE**	3,123	3,067,513	5,125.79	424,612	707.95	167,651	280.14	5.47
**SR**	3,051	805,900	6,358.78	199,527	1570.94	184,048	1,452.19	22.84
**2021**								
**YE**	3,040	4,245,102	7,093.53	410,317	684.12	159,229	266.07	3.75
**SR**	2,983	801,268	6,322.23	165,294	1301.41	109,146	861.19	13.62
**2022 (as of July 18)**								
**YE**	2,897	4,082,396	6,821.65	164,947	275.02	53,683	89.7	1.31
**SR**	2,824	848,319	6,693.48	96,293	758.14	49,815	393.05	5.87

*Hospitalized and death counts only include the patient records noted “Yes” in the hospital and death fields. All “missing” and “unknown” records are excluded.

Statistically significant differences were found between groups (YE 2020, YE 2021, YE 2022, SR 2020, SR 2021, and SR 2022) for incidence rates (F(5, 1854) = 39.87, *p* < .001), death rates (F(5, 1854) = 129.68, *p* < .001), and hospitalized rates (F(5, 1854) = 68.57, *p* < .001) (Table 2). A Tukey post hoc test indicated that the incidence rates in 2022 (YE22: 34.20±35.25; SR22: 33.56±29.74) were statistically significantly higher than in 2020 (YE20: 13.97±15.64; SR20: 17.33±17.93) and 2021 (YE21: 19.39±19.52; SR21: 17.28±17.76) for both groups. However, there was no statistically significant difference in incidence rates between YE and SR within any given year in the study.

**Table 2:** ANOVA table.

		Sum of Squares	df	Mean Square	F	Sig.
**Incidence rates**	Between Groups	94784.66	5	18956.93	39.87	< .001
	Within Groups	881566.37	1854	475.49		
	Total	976351.03	1859			
**Death rates**	Between Groups	3000.09	5	600.02	129.68	< .001
	Within Groups	8578.02	1854	4.63		
	Total	11578.10	1859			
**Hospitalization Rates**	Between Groups	2214.66	5	442.93	68.57	< .001
	Within Groups	11976.52	1854	6.46		
	Total	14191.18	1859			

Unlike the incidence rates, there were significant differences in hospitalized rates (hospitalized per 100,000 population) between YE and SR in 2020 (YE20: 1.93±1.54; SR20: 4.29±3.66), 2021 (YE21: 1.87±1.14; SR21: 2.57±2.72), and 2022 (YE22: 1.38±1.58; SR22: 3.81±3.59). However, there were no significant differences within either YE or SR in the studied years.

Likewise, there were significant differences in death rates (deaths per 100,000 population) between YE and SR in 2020 (YE20: 0.76±0.67; SR20: 3.06±3.65), 2021 (YE21: 0.73±0.53; SR21: 2.35±2.25), and 2022 (YE22: 0.45±0.70; SR22: 1.97±2.76). In addition, the death rates for SR in 2020 were significantly higher than in the following years. On the other hand, there was no significant difference for YE between 2020–2022.

### 3.2 Spatial dynamics of COVID-19 exposure disparities in the older adult groups

**Fig 3:**
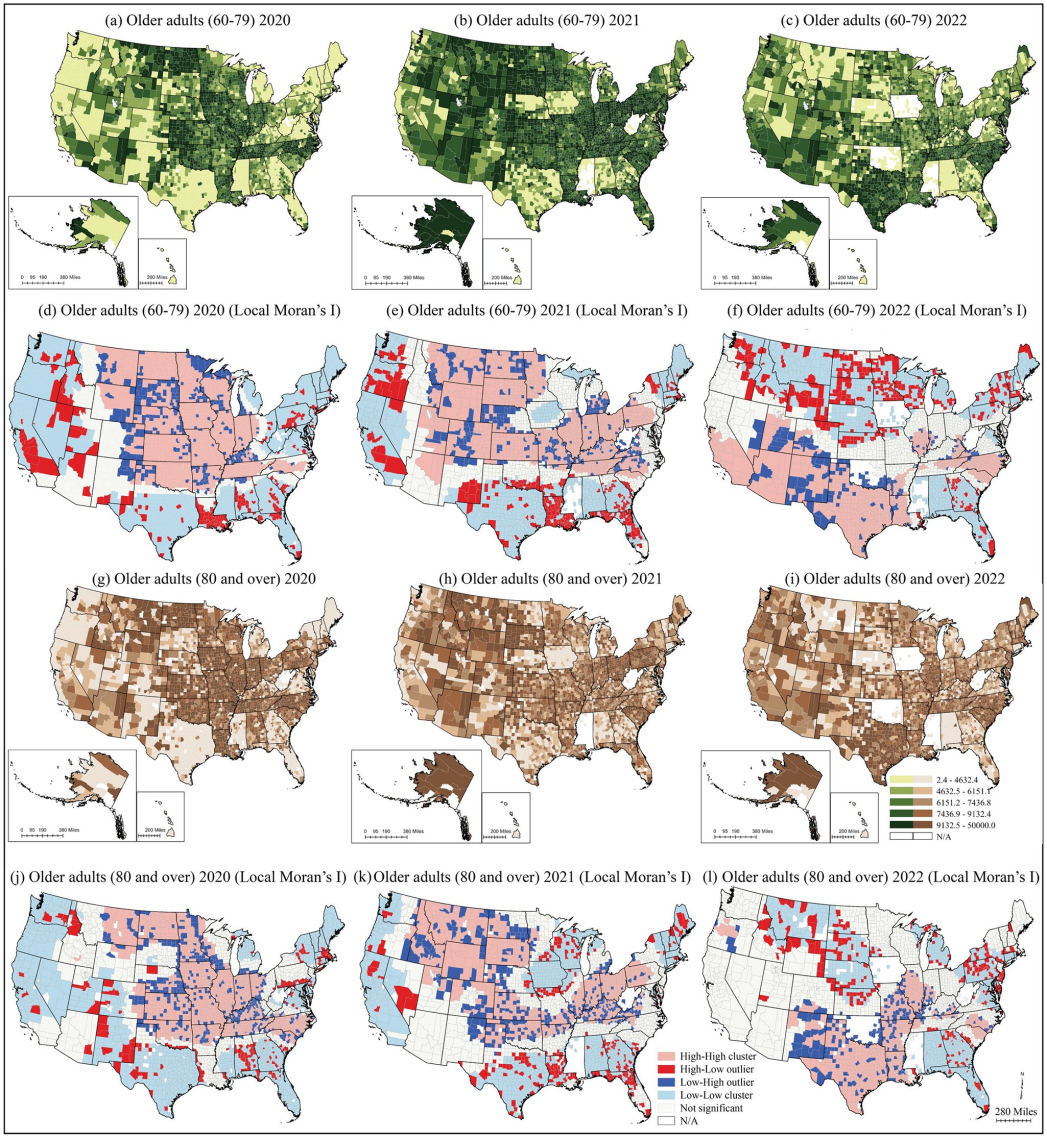
County-level COVID-19 incidence rates and local Moran’s I analysis results by year (2020, 2021, and 2022 as of July) for YE and SR. County and state boundaries are retrieved from the U.S. Census Bureau (https://www.census.gov/geographies/mapping-files/time-series/geo/carto-boundary-file.html). The shapefiles are released under the CC BY 4.0 license.

The spatial distribution of incidence rates varied across the states and years, but the county-level pattern difference between YE and SR was not prominent (Fig 3). Positive spatial autocorrelations were observed in incidence rates for the U.S. in all years and groups (Global Moran’s I: YE20: 0.26; SR20: 0.30; YE21: 0.17; SR21: 0.14; YE22: 0.17; SR 22: 0.14, *p*<0.001). The high-high cluster (concentrations of high incidence rates) in 2020 was located in Tennessee, North Carolina, and the Midwest states, with low outliers in South Dakota (Fig 3a, 3d, 3g and 3j). Later in the pandemic in 2021, the hotspot was mainly located in the Midwest (Fig 3b, 3e, 3h and 3k). The low-low cluster (concentrations of low incidence rates) in Iowa was probably due to previously unreported cases [39, 40]. The west coast, Southeast, and Northeast had low incidence rate clusters except for Louisiana. The pattern of the third wave shifted in 2022 (Fig 3c and 3i). The high-high clusters in YE appeared in the Southwest, Carolinas, and Illinois, with low incidence outliers in New Mexico, Utah, and Colorado (Fig 3f). However, the high-high cluster for SR was more concentrated in south-central states from Texas to Illinois and part of the Carolinas, while the low clusters were located in northcentral states, southeast, and northeast states (Fig 3l).

The shift of high-high clusters from the north (2020–21) to the south (2022) was attributable to a significant increase in COVID-19 incidence rates in some southern states, such as Texas, and a decrease in the area surrounding Montana. Before 2022, most of the counties in Texas had an incidence rate lower than 6151.1 per 100,000 population, while Montana had a rate higher than 9132.5 per 100,000 population. However, in 2022, the incidence rates in Texas counties mostly exceeded 9132.5 incidences per 100,000 population, whereas the area consisting of Montana, South Dakota, and Nebraska had apparent decreased incidence rates.

### 3.3 Determinants of health components

Fourteen components produced from 62 contextual variables on health determinants differentiated U.S. counties. The PCA explained roughly 68% of the variance in the input data among all 3142 U.S. counties (Table 3). Each component was named based on the variables that showed the most substantial loadings, typically those with values >0.7 or <-0.7. A detailed overview of these components, including their leading variables, is presented in Table 3, with in-depth descriptions available in S2 Table.

**Table 3:** Dimensions of determinants of health.

Factor	Name	% Variance Explained	Dominant Variables	Component Loading
**1**	Comorbidities and social status	20.709	Cardiovascular diseases (CVD)	0.923
			Stroke	0.918
			Hypertension	0.878
			Median household income	-0.751
**2**	Race, Political Affiliation and Chronic Diseases	6.978	Race—Non-white	0.738
			Democratic voters	0.722
			HIV	0.555
			Married population	-0.601
**3**	Healthcare Provider	6.616	Number of internal MDs	0.852
			Primary care providers	0.830
			Number of doctors	0.819
**4**	Healthcare Access	4.171	ICU beds	0.862
			Hospitals	0.702
			Emergency departments visits	0.667
			Telehealth services provided by hospitals	0.615
**5**	Social Capital	4.013	Religious affiliation	0.762
			Social associations	0.563
**6**	Natural Amenity	3.643	Natural Amenities Scale	0.828
			Access to Parks	0.517
**7**	Household Composition	3.629	Healthcare related occupation	0.529
			Health insurance	-0.500
			Households with children	-0.618
**8**	Air Quality	3.105	Ozone Days	0.690
			Particulate Matter Days	0.669
**9**	Urbanism	3.097	Population density	0.753
			Housing Units with No Car	0.709
**10**	Mobility	3.029	Grocery and pharmacy mobility change	-0.602
			Workplace mobility change	-0.713
**11**	Language and Culture	2.893	Language/ability to speak English (not well)	0.543
**12**	Mobile Clinics	2.232	Mobile van sites	-0.725
**13**	Environmental Hazards	1.972	Environmental hazards	0.706
**14**	Nursing Home	1.886	Nursing homes admissions	0.510
	***Total Variance Explained***	***67*.*971***		

### 3.4 Why were incidence rates different in older adult groups among counties?

To understand the variability in incidence rates among U.S. older adults, this study first employed correlation analysis using fourteen factors derived from PCA in relation to COVID-19 outcomes (Table 4). Only incidence rates were reported here due to concerns about the accuracy of fatality and hospitalization data. The overall incidence rate for both groups (YE and SR) between January 2020 and July 2022 was positively correlated with Factor 4-Healthcare access, Factor 5-Social capital, Factor 8-Air quality, and Factor 9-Urbanism. Among these factors, Factor 4 represents more ICU beds and high access to health services (Table 3), which allows higher testing capacity and more thorough case recording and reporting. Factor 5 speaks for religious gatherings, and Factor 8 represents high ozone concentration days and the amount of PM_2.5_. Factor 9- Urbanism, along with the most dominant variables, including population density and housing units with no vehicle ownership, signifies an increased risk of infection since residents staying in densely populated areas inherently face a greater likelihood of interpersonal contact, notably via public transportation systems [41]. Meanwhile, the exposures were negatively correlated with Factor 2-Race and communities, Factor 6-Natural amenity, Factor 7-Household composition, and Factor 10-Mobility. The negative effect of Factor 2- Race, Political Affiliation, and Chronic Diseases on older adults was confirmed by previous studies [42, 43], showing a higher percentage of White people getting infected and COVID-19 vaccine hesitancy decreasing more rapidly among Black individuals than among White individuals since December 2020. Green spaces decrease the risk of exposure to COVID-19. Moreover, the negative effects of Factor 7, dominated by more healthcare-related occupations, low health insurance, and low households with children, may be attributable to the introduction of COVID-19 vaccination among healthcare workers, and people with insurance also have more access to testing and diagnosis before developing a serious case of the disease. Factor 10-Mobility factor confirmed that higher mobility-based exposures increase the chance of getting infected.

**Table 4:** Correlations between COVID-19 incidence rates and determinants of health components by year among older adult groups between January 2020–July 2022.

Components	Overall (n = 3125)	YE20 (n = 3123)	YE21 (n = 3040)	YE22 (n = 2897)	SR20 (n = 3051)	SR21 (n = 2983)	SR22 (n = 2824)
**Factor 1_Comorbidities and Social Status**	.028	-.005	.051**	.071**	.010	.084**	.037
**Factor 2_Race, Political Affiliation, and Chronic Diseases**	-.104**	-.095**	-.217**	.103**	-.151**	-.214**	-.003
**Factor 3_Healthcare Provider**	.051**	-.082**	.041*	.182**	.002	.042*	.138**
**Factor 4_Healthcare Access**	.113**	.130**	.145**	.016	.086**	.110**	.007
**Factor 5_Social Capital**	.146**	.308**	.011	.077**	.172**	-.031	.078**
**Factor 6_Natural Amenity**	-.183**	-.301**	-.174**	.002	-.310**	-.090**	-.027
**Factor 7_Household Composition**	-.130**	-.098**	-.083**	-.103**	-.027	-.125**	-.078**
**Factor 8_Air Quality**	.114**	.049**	.032	.260**	.082**	.030	.243**
**Factor 9_Urbanism**	.127**	.145**	.141**	-0.015	.163**	.065**	-.002
**Factor 10_Mobility**	-.112**	-.213**	-.119**	.062**	-.207**	-.139**	-.044*
**Factor 11_Language and Culture**	.005	.034	-.134**	.136**	-.051**	-.084**	.083**
**Factor 12_Mobile Clinics**	.011	-.087**	.082**	.049**	.031	.050**	.033
**Factor 13_Environmental Hazards**	.039*	.024	.128**	-.063**	.084**	.114**	-.066**
**Factor 14_Nursing Home**	-.096**	-.124**	-.054**	-.022	-.083**	-.036	-.056**

**. *p* < 0.01 (2-tailed);

*. *p* <0.05 (2-tailed)

In 2020, incidence rates in both groups remained significantly and positively correlated with Factor 4-Healthcare access, Factor 5-Social capital, and Factor 9- Urbanism. Meanwhile, the incidence rates were significantly negatively correlated with Factor 2-Race and communities, Factor 6-Natural amenity, Factor 10-Mobility, and Factor 14-Nursing home. Unexpectedly, Factor 14 was significantly negatively correlated with COVID-19 incidence rates in older adults. However, from April to November 2020, observed declining mortality rates within nursing homes hint at several potential underlying mechanisms [44]. These may encompass advancements in clinical management within nursing facilities, enhanced availability and utilization of personal protective equipment, and genetic mutations of the virus [15, 44]. In addition, Factor 7- Household composition was significantly and negatively correlated with incidence rates for YE, but not significant for SR.

In 2021, most of the factors showed the same impacts on incidence rates as in the year 2020 for both groups. However, Factor 5-Social capital became insignificant for older adult groups, while Factor 7-Household composition and Factor 11-Language and culture became significantly negative for both groups. The negative correlation between incidence rates and low ability of English-speaking corresponds to the high incidences among Whites in 2021 [42, 44]. What is more, the result showed that Factor 13-Environmental hazards, dominated by the Toxics Release Inventory, had significant side effects on human health, increasing infection risk.

In 2022, Factor 3-Healthcare Provider became positive in the correlation, as did Factor 11-Language and Culture. The results suggested that language and culture may be obstacles to receiving appropriate healthcare services. The positive correlations with Factor 8-Air Quality in both groups confirmed the adverse effect of exposure to air pollution [45].

To further assess the relationship between pre-existing determinants of health and COVID-19 incidence rates, OLS were conducted. These models showed weak to moderate significance for incidence rates for YE and SR based on 14 components in 2020 (*R*_*YE*_^*2*^ = .25, *R*_*SR*_^*2*^ = .18), 2021 (*R*_*YE*_^*2*^ = .14, *R*_*SR*_^*2*^ = .11), and 2022 (*R*_*YE*_^*2*^ = .11, *R*_*SR*_^*2*^ = .07) (*p*<0.001) (S3 Table). For YE, all factors were significant in 2020 except for Factor 1-Comorbidities and social status and Factor 13-Environmental hazards. The relative importance of Factor 5-Social capital and Factor 6-Natural amenity was higher than other factors. In 2021, Factor 3-Healthcare provider and Factor 5-Social capital, Factor 8-Air quality, and Factor 12-Mobile clinics were no longer significant for the YE group, while Factor 2-Race, political affiliation and chronic diseases and Factor 11-Language and culture became the most important factors. In 2022, Factor 1 showed significance in the incidence rates, but Factor 4-Healthcare access, Factor 6, and Factor 9-Urbanism were not significant for the YE group. Among the SR group, Factors 1, 3, 7, and 12 were not significant in 2020, and the most significant factor was Factor 6. In the following year, Factors 7 and 12 showed more significance but Factors 1 and 3 stayed the same, and Factors 2 and 7 were dominant variables. However, in the year 2022, Factor 2 was no longer significant, nor were Factors 6, 9, and 10.

Positive spatial correlations were observed between the incidence rates and 14 components for all years (S4 Table). Based on Lagrange multiplier tests and spatial model residuals, the spatial error model best fits YE20 (*R*^*2*^ = .67), YE21 (*R*^*2*^ = .53), and SR20 (*R*^*2*^ = .44), while the spatial lag model is more optimal for the YE22 (*R*^*2*^ = .51), SR21 (*R*^*2*^ = .22), and SR22 (*R*^*2*^ = .28) (*p*<0.001). For YE, compared to the OLS model, Factor 1-Comorbidities and social status and Factor 13-Environmental hazards became significant in the spatial error model in 2020. However, Factors 10 and 12 were no longer significant when considering the spatial effects. In 2021, the COVID-19 incidence rates were significantly correlated with Factors 1, 2, 4–12 when considering the spatial effect. However, factors 13 and 14, which were significant in the OLS model, were no longer significant. In 2022, the incidence rates in YE were significantly and positively correlated with factors 2–4, 8, and 10–12, and negatively correlated with factors 7, 13, and 14 in the spatial lag model. For SR, the incidence rates in 2020 were positively and significantly correlated with factors 1,4,5,8, and 9, while negatively and significantly related with factors 2, 6, 10, and 14. The dominant variable was Factor 6-Natural amenity. In 2021, only Factors 4 and 9 were positively significant in the model, while Factors 2, 6, 7, 10, and 11 showed negatively significant effects. In 2022, Factor 8-Air quality became the dominant variable and positively correlated with the incidence rates for SR. The components had better prediction performance for YE than SR. However, none of the models passed the Breusch-Pagan test for heteroskedasticity, which means that although the spatial correlation of the error terms enhanced the model fit, it did not eliminate the spatial effects.

## 4. Discussion

Using a large dataset of over 13 million COVID-19 patients aged 60 years or older in the U.S., this study identified temporal, spatial, and demographic nonstationary dynamics of contextual variables on health within older adult groups. Geographically, the spatial clustering of high incidence rates for older adults shifted from the Midwest at the beginning of the pandemic in 2020 to the Southwest in 2022.

The results indicated that YE and SR had similar COVID-19 incidence rates in the contiguous U.S., but population aged 80 and over had much higher hospitalization rates, death rates, and case-fatality rates during the study period. Older adults, particularly those aged 65 years and above, have been more vulnerable to severe COVID-19 outcomes. By November 2022, this age group accounted for 92% of all COVID-19 related deaths in the U.S. [46]. This has largely been attributed to age being the most powerful risk factor for COVID-19, as well as the presence of other health conditions like chronic diseases and a weakened immune system. However, this study identified a nonstationary effect of COVID-19 in these older populations and found that although COVID-19 incidence rates were similar between individuals 60–79 years old and individuals older than 79, hospitalization and mortality rates were much higher in the latter group. This result can be associated with several factors like immunosenescence, which is a progressive decline of the immune system due to natural aging processes [47]. Immunosenescence leads to a decreased ability to fight infections and a lower response to vaccination in older adults. Furthermore, aging is associated with a higher prevalence of a pro-inflammatory state termed "inflammaging", characterized by a chronic, low-grade inflammation that can promote severe disease progression when an older adult contracts an infection like COVID-19. Moreover, older populations, especially those aged 79 and above, are more likely to have one or more chronic illnesses such as heart disease, lung disease, diabetes, or kidney disease. Those comorbidities have been recognized as amplifiers for the severity of COVID-19, exacerbating risks of hospitalization and mortality [48–50]. Additionally, these individuals are frequently found in nursing homes or other extended-care facilities, where there have been higher rates of transmission and severe outcomes due to challenges in infection control, shared living spaces, and a high prevalence of underlying conditions among residents. These factors might, singly or in combination, explain the higher hospitalization and mortality rates among individuals older than 79 years in comparison to those aged 60–79, even when the incidence rates of COVID-19 were the same in both groups.

The demographic and temporal nonstationary effects were confirmed by the fact that the relationships between determinants of health components and incidence rates varied across age groups and years. For example, Factor 2- race, political affiliations, and chronic diseases—was negatively and significantly correlated with incidence rates in both groups in 2020 and 2021, but it was positively and significantly associated with incidence rates for YE and was not significantly associated for SR in 2022.

Surprisingly, comorbidities and social status were not strongly correlated with the incidence rates for both groups, contradicting previous research [51]. However, when considering the spatial effects of the regression models, comorbidities and social status showed higher significance with the incidence rates in the first and second years of the pandemic for both groups. In addition, Factors 4-Healthcare access, 5-Social capital, 6-Natural amenity, 7-Household composition, 9-Urbanism, and 10-Mobility had stronger correlations with the incidence rates for both groups, in line with previous studies [27, 28, 30, 52–56]. Among all factors, healthcare provider and access, social capital, urbanism, and language and culture showed stronger effects for YE, while environmental hazards had more significant impacts for SR.

This study has some limitations. First, the missing data in West Virginia, Iowa, Louisiana, and Oklahoma for 2021–2022 may affect the results of the COVID-19 incidence pattern. Second, the correlation coefficients between determinants of health factors and COVID-19 incidence rates were relatively weak. This may be due to the use of the compounding effects of multiple variables to measure a single outcome. However, the factors explained a high portion of the variance change in the spatial models. Third, the study did not examine the pattern at a finer level such as the census tract. Future research based on the results of this study could look deeper at a finer geographic unit. Additionally, despite the utility of PCA, this method is sensitive to variable scales and may not fully capture the heterogeneity in local areas. Future analyses should incorporate more sensitivity analyses conducted at varying levels of granularity to ensure the stability and robustness of the main components. These limitations indicate that studies of COVID-19 exposure among older adults should adopt an interdisciplinary approach, combining infectious disease epidemiology, environmental epidemiology, and spatial epidemiology [33, 57, 58]. Further research is needed to deepen the understanding of how political, social, behavioral, environmental, and healthcare access factors relate to COVID-19 incidence, fatality, and hospitalization in the U.S. and across the world.

One unique strength of this study is the large sample size of COVID-19 patients aged 60 and over across the U.S. (over 13 million), ensuring sufficient statistical power for analysis. Second, the spatial disparities were exhibited by hotspot maps, showing the differences between YE and SR in 2020–2022. Third, a comprehensive list of determinants of health was created and 14 main components were generated, explaining 68% of the total variance. Furthermore, these 14 components explained 22–67% incidence rate variation with the spatial lag/error models, which had moderate to high statistical power. Lastly, the concept of demographic nonstationarity has been affirmed, and its existence in contextual variables related to COVID-19 incidence rates for older adults was confirmed.

The risk levels associated with COVID-19 infections vary considerably across the U.S., and their relationships with political, social, behavioral, environmental, and healthcare access factors are spatially, temporally, and demographically nonstationary, suggesting that a consistent stimulus might not necessarily result in a uniform shift in COVID-19 exposure risk. It is clear that older individuals may face more significant health risks from illnesses like COVID-19 due to physiological changes and pre-existing conditions, but those conditions do not affect the population equally along the demographic gradient across time and space [59]. Policies should ensure that these individuals have access to both primary and emergency healthcare. This could include enhancing home-based healthcare services or improving both the private and public transportation options (e.g., Medicaid transportation) for the older population, particularly for individuals aged 80 and over. Though telehealth has the potential to bridge the access gap, many older individuals have limited broadband access or may not be comfortable or familiar with digital technologies [60, 61]. Thus health policies could aim to improve digital literacy and expand the use of telehealth services. Furthermore, the pandemic has highlighted the vulnerabilities of older adults living in long-term care facilities such as nursing homes [62]. Policies should be implemented to enhance the safety measures in these facilities, including regular testing for infectious diseases, improved sanitation protocols, and training for staff on infectious disease management. In addition, the high mortality rates from COVID-19 among older adults, particularly those with comorbidities, underscores the importance of health promotion and disease prevention strategies. Interventions should adopt place- and age-based perspectives to address specific needs and conditions. Different recovery and mitigation measures, not only for COVID-19 but for other health conditions, should consider different population structures instead of implementing one-fit-all policies.

## Supporting information

S1 Table Variable names and descriptions. (DOCX)

S2 Table Detailed table for dimensions of determinants of health. (DOCX)

S3 Table Ordinary least squares: COVID-19 incidence rates with fourteen determinants of health components. (DOCX)

S4 Table Spatial lag/error model: COVID-19 incidence rates with fourteen determinants of health components. (DOCX)

S1 Data (XLSX)
