# Supplementary material for: Spatial, temporal, and demographic nonstationary dynamics of COVID-19 exposure among older adults in the U.S
Source: PLoS One. 2024 Aug 22;19(8):e0307303. doi: 10.1371/journal.pone.0307303 (PMC11341038; doi:10.1371/journal.pone.0307303)
Supplement: S2 Table — (DOCX) [file pone.0307303.s002.docx]

**S2 Table****. Detailed Table for Dimensions of Determinants of Health**

| Factor | Name | % Variance Explained | Dominant Variables | Component Loading | Description |
| --- | --- | --- | --- | --- | --- |
| 1 | Comorbidities and Social Status | 20.709 | Cardiovascular diseases (CVD) | 0.923 | Comorbidities are associated with COVID-19 infections and severe COVID-19 illness (Fang et al 2020). Obesity, diabetes with complications, and anxiety had the strongest association with death (CDC 2020a). Low social status is perceived as a factor in not following the social distancing order and mask mandates, but consideration must be taken on that low median house income is a vital contributor to COVID incidence as fewer resources are available. |
|  |  |  | Stroke | 0.918 |  |
|  |  |  | Hypertension | 0.878 |  |
|  |  |  | Diabetes | 0.869 |  |
|  |  |  | Mental Health | 0.869 |  |
|  |  |  | Physical inactivity | 0.852 |  |
|  |  |  | Below Poverty | 0.852 |  |
|  |  |  | Unemployment | 0.802 |  |
|  |  |  | Educational attainment – below college | 0.728 |  |
|  |  |  | Obesity | 0.709 |  |
|  |  |  | Asthma | 0.650 |  |
|  |  |  | Female Headed Households | 0.648 |  |
|  |  |  | Population with disability | 0.634 |  |
|  |  |  | Low Birth Weight | 0.616 |  |
|  |  |  | Medically Underserved Areas/population | 0.535 |  |
|  |  |  | Depression | 0.532 |  |
|  |  |  | Food environment index | -0.695 |  |
|  |  |  | Alcohol | -0.730 |  |
|  |  |  | Median income | -0.751 |  |
| 2 | Race, Political Affiliation, and Chronic Diseases | 6.978 | Race - Non-white | 0.738 | African American populations had 1.1 times greater rate of SARS-Cov-2 infections risk than Whites, and Hispanics or Latino had 1.5 times a greater number of cases (CDC 2020a). The HIV positive population was heavily affected during COVID-19 since it was harder to receive treatment and monitoring during this time (Msomi et al. 2021). |
|  |  |  | Democratic voters | 0.722 |  |
|  |  |  | HIV | 0.555 |  |
|  |  |  | Female Headed Households | 0.540 |  |
|  |  |  | Depression | -0.535 |  |
|  |  |  | Cancer (excluding skin cancer) | -0.557 |  |
|  |  |  | Married population | -0.601 |  |
| 3 | Healthcare Provider | 6.616 | Number of internal MDs | 0.852 | Highlight those counties where sufficient healthcare services might be expected. These two factors emphasize the “availability” and “accessibility” of Penchansky’s 5 “A’s” model. The “Availability” is the extent to which facilities have the resources (personnel, number of beds, etc.) to meet the patient’s needs. “Accessibility” is the relationship between the location of those facilities and the location of the patients (Penchansky & Thomas 1981). |
|  |  |  | Primary care providers | 0.830 |  |
|  |  |  | Number of doctors | 0.819 |  |
|  |  |  | Rural population | -0.539 |  |
| 4 | Healthcare Access | 4.171 | ICU beds | 0.862 |  |
|  |  |  | Hospitals | 0.702 |  |
|  |  |  | Emergency departments visits | 0.667 |  |
|  |  |  | Telehealth service provided by hospitals | 0.615 |  |
| 5 | Social Capital | 4.013 | Religious affiliation | 0.762 | Religions require the faithful to congregate (Tan et al. 2022), but perceptions of high neighborhood social capital are related to more positive outcomes in many dimensions: increasing personal resilience, ability to cope with uncertainty, perceptions of community solidarity, and reported compliance with public health measures (Carter & Cordero 2022). |
|  |  |  | Social associations | 0.563 |  |
| 6 | Natural Amenity | 3.643 | Natural Amenities Scale | 0.828 | Green spaces, like parks, and better environment have become one of the only sources of resilience amidst the COVID pandemic because of their positive effects on physical, psychological, and spiritual wellness (Geng et al. 2021). |
|  |  |  | Access to Parks | 0.517 |  |
| 7 | Household Composition | 3.629 | Healthcare related occupation | 0.529 | High exposure and require more routine testing, leading to a high number of reported cases including mild or asymptomatic cases. People with insurance also have more access to testing and diagnosis before getting a serious case of the virus. |
|  |  |  | Health insurance | -0.500 |  |
|  |  |  | Households with children | -0.618 |  |
| 8 | Air Quality | 3.105 | Ozone Days | 0.690 | The risks of chronic exposure to air pollution have been confirmed (Schraugnagel 2020). |
|  |  |  | Particulate Matter Days | 0.669 |  |
| 9 | Urbanism | 3.097 | Population density | 0.753 | High population density means a higher probability to come into contact with others (Bhadra et al. 2021). A high percentage of housing units without car ownership indicate a high use of public transportation (Shen et al. 2020). |
|  |  |  | Housing Units with No Car | 0.709 |  |
| 10 | Mobility | 3.029 | Grocery and pharmacy mobility change | -0.602 | Higher mobility-based exposures increase the chance of getting infected, while the negative-loading mobility change could decrease the exposure opportunities (Huang & Kwan 2022). |
|  |  |  | Workplace mobility change | -0.713 |  |
| 11 | Language and Culture | 2.893 | Language/ability to speak English (not well) | 0.543 | Structural forces limit access to health care and systemic racism and discrimination toward the poor English speakers can make it difficult to develop trusting relationships with healthcare providers (Curtice & Choo 2020). |
| 12 | Mobile Clinics | 2.232 | Mobile van sites | -0.725 | Provide access to healthcare, especially for displaced and isolated individuals; Offer flexibility in the setting of inadequate healthcare infrastructure, and fill gaps in the healthcare safety net (Attipoe-Dorcoo et al. 2020) . |
| 13 | Environmental Hazards | 1.972 | Environmental hazards | 0.706 | Toxic emissions have significant side effects on human health, increasing the risk for cardiovascular, endocrine, nervous system, and respiratory disease. |
| 14 | Nursing Home | 1.886 | Nursing homes admissions | 0.510 | Close contact with nursing home staff, social gatherings with other residents, and low protection from pathogens contribute to a high-risk of getting infected and severe consequences (Kittang et al. 2020). |
|  | ***Total Variance Explained*** | ***67.971*** |  |  |  |

Note: Only those variables with component loadings of +/- 0.50 or higher/lower are reported here.
