# Supplementary material for: Spatial, temporal, and demographic nonstationary dynamics of COVID-19 exposure among older adults in the U.S
Source: PLoS One. 2024 Aug 22;19(8):e0307303. doi: 10.1371/journal.pone.0307303 (PMC11341038; doi:10.1371/journal.pone.0307303)
Supplement: S3 Table — (DOCX) [file pone.0307303.s003.docx]

**S3 Table****. Ordinary Least Squares: COVID-19 Incidence Rates with Fourteen Determinants of Health Components**

| Variable | Coefficient | Std. Error | t | p | Coefficient | Std. Error | t | p | Coefficient | Std. Error | t | p |
| --- | --- | --- | --- | --- | --- | --- | --- | --- | --- | --- | --- | --- |
| Older Adults (60-79) 2020 | | | | | **Older Adults (60-79) 2021** | | | | **Older Adults (60-79) 2022** | | | |
| Moran's I | 49.77 (p<0.001) | | | | 43.18 (p<0.001) | | | | 42.73 (p<0.001) | | | |
| CONSTANT | 5926.59 | 48.40 | 122.45 | 0.00 | 7472.65 | 45.83 | 163.04 | 0.00 | 6216.76 | 47.87 | 129.87 | 0.00 |
| Factor 1 | -0.71 | 48.64 | -0.01 | 0.99 | 62.91 | 47.35 | 1.33 | 0.18 | 123.43 | 50.09 | 2.46 | 0.01 |
| Factor 2 | -183.58 | 48.79 | -3.76 | 0.00 | -475.83 | 47.23 | -10.08 | 0.00 | 204.97 | 48.80 | 4.20 | 0.00 |
| Factor 3 | -305.99 | 48.46 | -6.31 | 0.00 | -38.68 | 45.80 | -0.84 | 0.40 | 256.14 | 47.27 | 5.42 | 0.00 |
| Factor 4 | 478.59 | 58.91 | 8.12 | 0.00 | 335.83 | 55.42 | 6.06 | 0.00 | -16.01 | 58.97 | -0.27 | 0.79 |
| Factor 5 | 855.70 | 51.11 | 16.74 | 0.00 | 73.54 | 48.10 | 1.53 | 0.13 | 114.88 | 50.90 | 2.26 | 0.02 |
| Factor 6 | -887.30 | 48.54 | -18.28 | 0.00 | -400.32 | 45.56 | -8.79 | 0.00 | 70.40 | 47.43 | 1.48 | 0.14 |
| Factor 7 | -268.75 | 49.72 | -5.41 | 0.00 | -315.10 | 46.78 | -6.74 | 0.00 | -281.77 | 48.43 | -5.82 | 0.00 |
| Factor 8 | 120.38 | 49.69 | 2.42 | 0.02 | -40.98 | 46.27 | -0.89 | 0.38 | 513.80 | 47.43 | 10.83 | 0.00 |
| Factor 9 | 245.99 | 68.19 | 3.61 | 0.00 | 402.42 | 65.19 | 6.17 | 0.00 | 41.00 | 66.79 | 0.61 | 0.54 |
| Factor 10 | -557.44 | 49.32 | -11.30 | 0.00 | -262.99 | 46.74 | -5.63 | 0.00 | 261.07 | 48.84 | 5.35 | 0.00 |
| Factor 11 | 113.38 | 51.38 | 2.21 | 0.03 | -449.92 | 48.18 | -9.34 | 0.00 | 294.55 | 49.74 | 5.92 | 0.00 |
| Factor 12 | -182.55 | 48.28 | -3.78 | 0.00 | 70.26 | 45.21 | 1.55 | 0.12 | 187.78 | 46.59 | 4.03 | 0.00 |
| Factor 13 | -7.58 | 48.93 | -0.15 | 0.88 | 123.29 | 45.86 | 2.69 | 0.01 | -223.67 | 47.14 | -4.74 | 0.00 |
| Factor 14 | -307.79 | 49.22 | -6.25 | 0.00 | -132.92 | 47.64 | -2.79 | 0.01 | -149.74 | 49.04 | -3.05 | 0.00 |
| Older Adults (80 and over) 2020 | | | | | **Older Adults (80 and over) 2021** | | | | **Older Adults (80 and over) 2022** | | | |
| Moran's I | 34.88 (p<0.001) | | | | 27.42 (p<0.001) | | | | 42.73 (p<0.001) | | | |
| CONSTANT | 7327.20 | 71.99 | 101.78 | 0.00 | 7090.88 | 56.23 | 126.10 | 0.00 | 6825.93 | 56.39 | 121.04 | 0.00 |
| Factor 1 | 59.56 | 73.22 | 0.81 | 0.42 | 81.41 | 58.84 | 1.38 | 0.17 | 102.79 | 59.82 | 1.72 | 0.09 |
| Factor 2 | -605.53 | 73.32 | -8.26 | 0.00 | -527.53 | 58.46 | -9.02 | 0.00 | -41.56 | 57.66 | -0.72 | 0.47 |
| Factor 3 | -116.58 | 72.21 | -1.61 | 0.11 | -105.17 | 56.47 | -1.86 | 0.06 | 178.80 | 57.05 | 3.13 | 0.00 |
| Factor 4 | 461.61 | 87.45 | 5.28 | 0.00 | 321.50 | 67.84 | 4.74 | 0.00 | -95.53 | 69.43 | -1.38 | 0.17 |
| Factor 5 | 760.83 | 77.28 | 9.84 | 0.00 | 55.60 | 59.66 | 0.93 | 0.35 | 274.87 | 60.28 | 4.56 | 0.00 |
| Factor 6 | -1259.28 | 73.95 | -17.03 | 0.00 | -166.35 | 56.59 | -2.94 | 0.00 | -33.00 | 56.71 | -0.58 | 0.56 |
| Factor 7 | -9.09 | 75.91 | -0.12 | 0.90 | -552.68 | 58.47 | -9.45 | 0.00 | -268.67 | 58.29 | -4.61 | 0.00 |
| Factor 8 | 181.86 | 74.66 | 2.44 | 0.01 | -165.31 | 57.27 | -2.89 | 0.00 | 518.91 | 56.48 | 9.19 | 0.00 |
| Factor 9 | 426.97 | 102.14 | 4.18 | 0.00 | 245.16 | 80.60 | 3.04 | 0.00 | 63.11 | 78.56 | 0.80 | 0.42 |
| Factor 10 | -700.13 | 73.47 | -9.53 | 0.00 | -323.81 | 57.00 | -5.68 | 0.00 | 32.78 | 57.42 | 0.57 | 0.57 |
| Factor 11 | -142.67 | 78.91 | -1.81 | 0.07 | -475.73 | 61.43 | -7.74 | 0.00 | 180.26 | 60.65 | 2.97 | 0.00 |
| Factor 12 | -80.61 | 73.36 | -1.10 | 0.27 | -161.49 | 56.68 | -2.85 | 0.00 | 216.76 | 80.99 | 2.68 | 0.01 |
| Factor 13 | 153.89 | 72.65 | 2.12 | 0.03 | 126.01 | 56.15 | 2.24 | 0.02 | -313.08 | 55.56 | -5.63 | 0.00 |
| Factor 14 | -179.89 | 74.84 | -2.40 | 0.02 | -145.63 | 58.55 | -2.49 | 0.01 | -174.11 | 58.18 | -2.99 | 0.00 |

Factor 1- Comorbidities and Social Status, Factor 2- Race and Political Affiliation, Factor 3- Healthcare Provider, Factor 4- Healthcare Access, Factor 5- Social Capital, Factor 6- Natural Amenity, Factor 7- Household Composition, Factor 8- Air Quality, Factor 9- Urbanism, Factor 10- Mobility, Factor 11- Language and Culture, Factor 12- Mobile Clinics, Factor 13- Environment, and Factor 14- Nursing Home.
