# Supplementary material for: Spatial, temporal, and demographic nonstationary dynamics of COVID-19 exposure among older adults in the U.S
Source: PLoS One. 2024 Aug 22;19(8):e0307303. doi: 10.1371/journal.pone.0307303 (PMC11341038; doi:10.1371/journal.pone.0307303)
Supplement: S4 Table — (DOCX) [file pone.0307303.s004.docx]

**S4 Table****. Spatial Lag/Error Model: COVID-19 Incidence Rates with Fourteen Determinants of Health Components**

| Variable | Coefficient | Std. Error | z | p | Coefficient | Std. Error | z | p | Coefficient | Std. Error | z | p |
| --- | --- | --- | --- | --- | --- | --- | --- | --- | --- | --- | --- | --- |
|  | **Older Adults (60-79) 2020** | | | | **Older Adults (60-79) 2021** | | | | **Older Adults (60-79) 2022** | | | |
| *Model (R^2^)* | Spatial error (0.67) | | | | Spatial error (0.53) | | | | Spatial Lag (0.51) | | | |
| W_Dependent variable |  |  |  |  |  |  |  |  | 0.69 | 0.02 | 44.77 | 0.00 |
| CONSTANT | 5727.62 | 159.98 | 35.80 | 0.00 | 7219.82 | 125.01 | 57.76 | 0.00 | 1914.96 | 102.41 | 18.70 | 0.00 |
| Factor 1 | 527.75 | 66.69 | 7.91 | 0.00 | 197.01 | 66.37 | 2.97 | 0.00 | 52.55 | 37.15 | 1.41 | 0.16 |
| Factor 2 | 215.17 | 53.34 | 4.03 | 0.00 | -252.09 | 54.47 | -4.63 | 0.00 | 212.39 | 36.40 | 5.84 | 0.00 |
| Factor 3 | -163.37 | 38.12 | -4.29 | 0.00 | -25.17 | 39.16 | -0.64 | 0.52 | 275.19 | 35.10 | 7.84 | 0.00 |
| Factor 4 | 162.66 | 43.75 | 3.72 | 0.00 | 135.70 | 45.29 | 3.00 | 0.00 | 153.84 | 43.77 | 3.51 | 0.00 |
| Factor 5 | 498.05 | 60.18 | 8.28 | 0.00 | 204.76 | 59.56 | 3.44 | 0.00 | 5.39 | 37.76 | 0.14 | 0.89 |
| Factor 6 | -788.35 | 69.16 | -11.40 | 0.00 | -665.13 | 67.60 | -9.84 | 0.00 | -55.83 | 35.18 | -1.59 | 0.11 |
| Factor 7 | -248.83 | 52.06 | -4.78 | 0.00 | -122.75 | 52.26 | -2.35 | 0.02 | -201.39 | 36.06 | -5.58 | 0.00 |
| Factor 8 | 352.95 | 55.16 | 6.40 | 0.00 | 241.85 | 54.90 | 4.41 | 0.00 | 224.34 | 35.84 | 6.26 | 0.00 |
| Factor 9 | 166.75 | 62.76 | 2.66 | 0.01 | 349.63 | 64.65 | 5.41 | 0.00 | 6.80 | 49.55 | 0.14 | 0.89 |
| Factor 10 | -64.12 | 43.25 | -1.48 | 0.14 | -221.28 | 44.16 | -5.01 | 0.00 | 93.14 | 36.47 | 2.55 | 0.01 |
| Factor 11 | 327.12 | 47.85 | 6.84 | 0.00 | -182.39 | 48.77 | -3.74 | 0.00 | 83.36 | 36.97 | 2.25 | 0.02 |
| Factor 12 | -35.82 | 36.71 | -0.98 | 0.33 | 119.69 | 37.62 | 3.18 | 0.00 | 148.23 | 34.57 | 4.29 | 0.00 |
| Factor 13 | -177.25 | 39.39 | -4.50 | 0.00 | -4.14 | 40.25 | -0.10 | 0.92 | -119.86 | 35.00 | -3.42 | 0.00 |
| Factor 14 | -163.63 | 38.25 | -4.28 | 0.00 | -42.64 | 40.45 | -1.05 | 0.29 | -143.08 | 36.37 | -3.93 | 0.00 |
| LAMBDA | 0.81 | 0.01 | 65.61 | 0.00 | 0.74 | 0.01 | 49.88 | 0.00 |  |  |  |  |
|  | **Older Adults (80 and over) 2020** | | | | **Older Adults (80 and over) 2021** | | | | **Older Adults (80 and over) 2022** | | | |
| *Model (R^2^)* | Spatial error (0.44) | | | | Spatial Lag (0.22) | | | | Spatial Lag (0.28) | | | |
| W_Dependent variable |  |  |  |  | 0.40 | 0.02 | 17.68 | 0.00 | 0.51 | 0.02 | 24.81 | 0.00 |
| CONSTANT | 7258.82 | 154.14 | 47.09 | 0.00 | 4236.94 | 168.92 | 25.08 | 0.00 | 3295.53 | 149.07 | 22.11 | 0.00 |
| Factor 1 | 306.30 | 105.51 | 2.90 | 0.00 | 65.81 | 54.74 | 1.20 | 0.23 | 56.67 | 52.26 | 1.08 | 0.28 |
| Factor 2 | -357.40 | 89.56 | -3.99 | 0.00 | -330.39 | 55.64 | -5.94 | 0.00 | 10.53 | 50.39 | 0.21 | 0.83 |
| Factor 3 | -70.54 | 67.29 | -1.05 | 0.29 | -96.79 | 52.57 | -1.84 | 0.07 | 198.85 | 49.85 | 3.99 | 0.00 |
| Factor 4 | 316.63 | 79.30 | 3.99 | 0.00 | 218.03 | 63.30 | 3.44 | 0.00 | 12.76 | 60.69 | 0.21 | 0.83 |
| Factor 5 | 559.80 | 98.02 | 5.71 | 0.00 | 56.46 | 55.54 | 1.02 | 0.31 | 161.82 | 52.78 | 3.07 | 0.00 |
| Factor 6 | -1188.09 | 108.35 | -10.97 | 0.00 | -133.34 | 52.87 | -2.52 | 0.01 | -85.41 | 49.55 | -1.72 | 0.08 |
| Factor 7 | -108.72 | 90.05 | -1.21 | 0.23 | -407.53 | 54.88 | -7.43 | 0.00 | -159.92 | 50.98 | -3.14 | 0.00 |
| Factor 8 | 301.39 | 93.20 | 3.23 | 0.00 | -57.08 | 53.37 | -1.07 | 0.28 | 339.31 | 50.15 | 6.77 | 0.00 |
| Factor 9 | 346.86 | 109.58 | 3.17 | 0.00 | 192.95 | 75.01 | 2.57 | 0.01 | 20.05 | 68.64 | 0.29 | 0.77 |
| Factor 10 | -362.69 | 75.50 | -4.80 | 0.00 | -244.08 | 53.42 | -4.57 | 0.00 | 13.21 | 50.18 | 0.26 | 0.79 |
| Factor 11 | 0.54 | 85.74 | 0.01 | 0.99 | -346.08 | 57.57 | -6.01 | 0.00 | 97.11 | 53.06 | 1.83 | 0.07 |
| Factor 12 | -61.03 | 67.07 | -0.91 | 0.36 | -103.14 | 52.84 | -1.95 | 0.05 | 222.74 | 70.76 | 3.15 | 0.00 |
| Factor 13 | -56.40 | 70.44 | -0.80 | 0.42 | 85.34 | 52.26 | 1.63 | 0.10 | -189.23 | 48.61 | -3.89 | 0.00 |
| Factor 14 | -141.14 | 71.29 | -1.98 | 0.05 | -73.05 | 54.63 | -1.34 | 0.18 | -118.13 | 50.84 | -2.32 | 0.02 |
| LAMBDA | 0.62 | 0.02 | 33.50 | 0.00 |  |  |  |  |  |  |  |  |

Factor 1- Comorbidities and Social Status, Factor 2- Race, Political Affiliation and Chronic Diseases, Factor 3- Healthcare Provider, Factor 4- Healthcare Access, Factor 5- Social Capital, Factor 6- Natural Amenity, Factor 7- Household Composition, Factor 8- Air Quality, Factor 9- Urbanism, Factor 10- Mobility, Factor 11- Language and Culture, Factor 12- Mobile Clinics, Factor 13- Environment, and Factor 14- Nursing Home.
